# Selective Hydrothermal Leaching of Aluminum from Al_3_YRh_*x*_ (*x* = 0, 0.2, 0.5, 1.0) Intermetallic Compounds: The Effect of Rh Variants in Comparing the Catalytic CO Oxidation and CO-PROX Reactions

**DOI:** 10.1021/acsmaterialsau.4c00140

**Published:** 2024-11-29

**Authors:** Balasubramanian Sriram, Sea-Fue Wang, Satoshi Kameoka

**Affiliations:** † Department of Materials and Mineral Resources Engineering, National Taipei University of Technology, No. 1, Section 3, Chung-Hsiao East Road, Taipei 106, Taiwan, ROC; ‡ Institute of Multidisciplinary Research for Advanced Materials, Tohoku University, 2-1-1 Katahira, Aoba-ku, Sendai 980-8577, Japan

**Keywords:** CO oxidation, CO-PROX, hydrothermal leaching, CO conversion, intermetallic compounds, environmental pollution

## Abstract

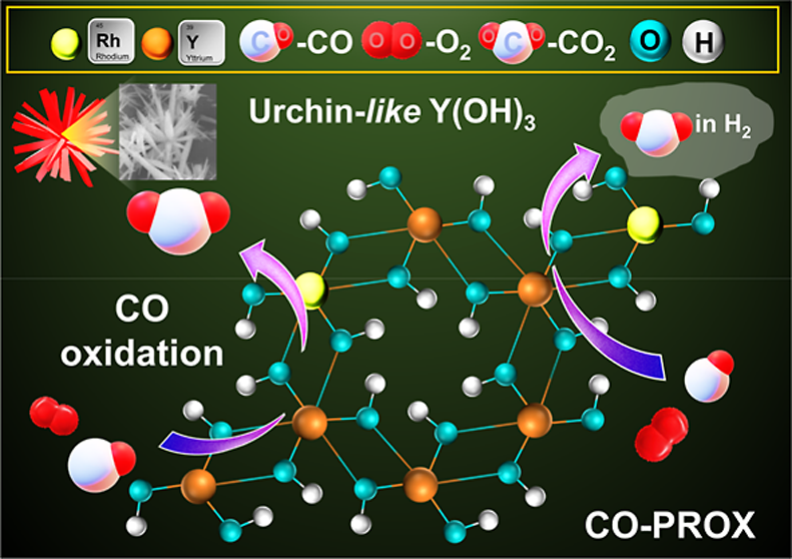

Wealth from modern civilization and globalization accelerates natural resource extraction and damages the Earth’s environment. Elevated mute assassin “carbon monoxide (CO)” levels impede aerobic life. We need to develop limiting technologies to overcome these constraints. Stern environmental agreements to reduce CO levels are significant. In this work, a hydrothermal leaching (HyTL) of Al_3_Y-Rh_*x*_ (*x* = 0, 0.2, 0.5, 1.0) intermetallic compounds yields Y(OH)_3_ products with well-distributed rhodium (Rh). The HyTL method and active Rh element improved HyTL Al_3_Y-Rh_0.5_ catalytic CO oxidation and the preferential oxidation of the CO (CO-PROX) performance. Metal-support interactions and HyTL Al_3_Y-Rh_0.5_ catalyst synergy produce oxygen vacancies, govern CO oxidation, and standardize oxygen mobility. This is essential to the synthesized catalyst’s improved catalytic performance. All low-temperature instances of Rh have strong catalytic activity. This study advances CO catalytic oxidation and CO-PROX over the HyTL Al_3_Y-Rh_0.5_ catalyst, ensuring the aggregation-activation process. The findings support an understanding of low-temperature catalytic systems.

## 1 Introduction

The rising use of fossil fuels has released poisonous chemicals into the atmosphere, which causes environmental problems. Air pollution from burning fossil fuels and byproducts is a significant health risk.^1^ The bionetwork imbalance is caused by “carbon monoxide (CO)” among other air contaminants. According to global atmospheric chemistry, CO is a universal reactive trace gas and an anthropogenic emission that causes greenhouse effects.^2,3^ CO depletes the ozone layer, causes respiratory issues, and raises global warming. CO exposure causes leaf curling and decolorization, early chlorophyll aging, decreased leaf size and main root length, reduced cellular respiration, and nitrogen fixation failure. Given those mentioned above, atmospheric CO significantly impacts plant development and physiology.^4^ The outcome is reduced agricultural productivity. During inhalation, it irreversibly binds to the Fe atom in hemoglobin (Hb), reducing oxygen transfer to body tissues.^5^ Hb to carry oxygen to the brain and other organs is diminished. Hypoxia injury can cause brain damage, decreased vision, delayed reflexes, lethargy, and even death.^6,7^ High levels of carbon monoxide can affect the brain activity for short periods of time. High amounts of CO in the air make air quality worse. CO is very dangerous to both people and animals. Exposure to 100 ppm of CO is bad for people’s health. Children are more likely to develop CO poisoning than adults. Because of this, the National Institute for Occupational Safety and Health (NIOSH) says that the CO levels should be lowered to 35 ppm. So, lowering or changing CO is still a big problem that needs to be fixed quickly.^8,9^

Catalytic CO oxidation and CO-PROX have garnered attention recently due to their potential industrial applications that remove CO from H_2_ for fuel cell use and CO gas sensors.^10,11^ The catalytic oxidation of CO at low temperatures is a primary scientific research concern. Scientists are studying rational designs for low-temperature nanocatalysts with well-defined shapes that can treat exhaust gases. Intermetallic compounds occur at lattice sites with precisely arranged stoichiometric compositions, which stimulates the interest of scientists in catalytic materials.^12,13^ Compared with other materials, this finding may point to catalytic properties that are better. Intermetallic systems are useful in heterogeneous catalytic processes because they can distribute important chemicals that can combine across material boundaries. Intermetallic molecules can achieve good selectivity, stability, and catalytic activity due to the way they couple with other metals. A lot of new, complex synthetic methods have been produced to improve the reach of intermetallic compounds and give more control over particle size, form, surface area, and morphology.^14,15^ Unfortunately, irrepressible aggregation is the most significant synthetic challenge. To solve this issue, conventional and hydrothermal leaching (HyTL) was used to synthesize promising candidates from intermetallic compounds.^13^ For instance, Umesh et al. 2022,^13^ used a single-phase Al–Ce intermetallic compound to leach Pt nanoparticles onto an AlCe platform in one step. The synthesized CeO_2_/Pt catalyzed CO oxidation exhibits at low temperatures. Kameoka et al. 2016,^16^ used a single-phase Al–Fe–Pt intermetallic compound to leach Pt nanoparticles onto Fe_3_O_4_ in one step. The synthesized porous matrix catalyzed the CO oxidation better. Similarly, Zielasek et al. 2006,^17^ leached nanoporous gold from Au–Ag solid solution to improve CO oxidation catalysis.

The well-ordered placement of atoms in intermetallic compounds renders them homogeneous and suitable for HyTL.^18^ Aluminum (Al)-based intermetallic compounds are preferred due to their low melting point, lower density, enhanced specific durability, accessibility, and large-scale production. Leaching enables amphoteric Al alloys to be flexible and functional in acidic and basic conditions.^19,20^ The present study extensively investigates Al_3_Y-Rh_*x*_ intermetallic compounds (*x* = 0, 0.2, 0.5, 1.0) and compares their enhanced catalytic activity. HyTL Al_3_Y-Rh_0.5_ endured high catalytic activity attributed to yttria’s abundant oxygen vacancies, high thermal stability, equilibrated acid–base sites, increased resistance at high operating temperatures and stress, and reduced oligomerization.^21−23^ Catalysis has been further emphasized by significant yttria electronic structure and shape insights. The sorption of carbon monoxide on yttrium hydroxide [Y(OH_3_)] showed that the phase of yttrium influences the CO interaction intensity. They have additionally studied the effect of rhodium (Rh) dopant on intermetallic compounds. The spotlight that has been placed on Rh’s rapid global growth has increased its use (81% of Rh) for catalyst production.^24^ In this way, Rh can be relied on extensively to improve structural stability and active site homogeneity, which boosts catalyst performance. No comparable element for Rh has been found due to its partially full 4d orbital, which aids CO oxidation and CO-PROX catalyst production.^25−27^

**Scheme 1:**
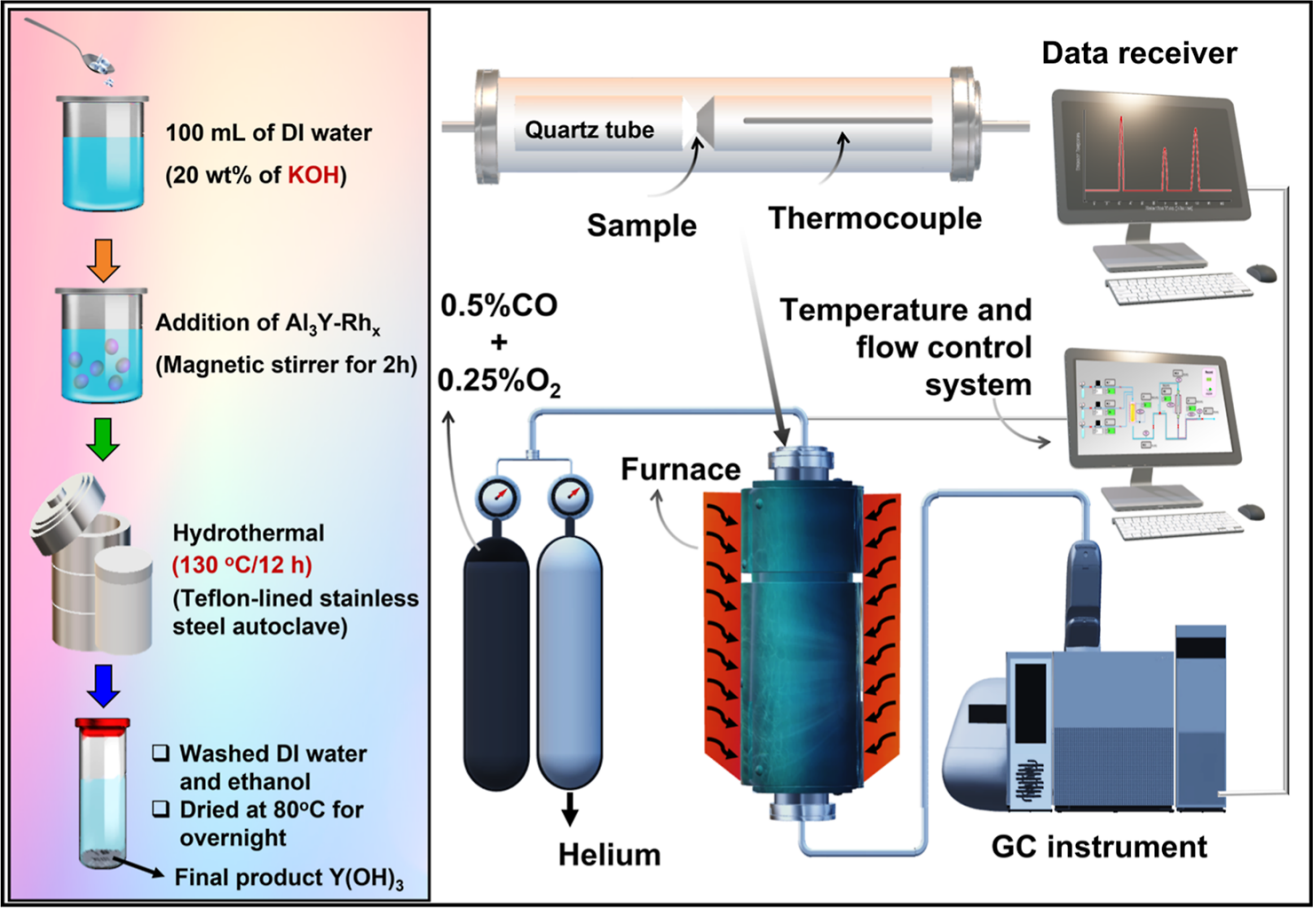
Schematic Illustration of the KOH HyTL of Al_3_Y-Rh_*x*_ Intermetallic Compounds Using CO Oxidation Catalytic Equipment

In this work, HyTL Al_3_Y-Rh_*x*_ intermetallic compound was utilized at 130 °C for 12 h for CO oxidation and CO-PROX (Scheme 1). Several characterization techniques were utilized to analyze leached samples, including X-ray diffraction (XRD), scanning electron microscopy (SEM), transmission electron microscopy (TEM), and energy dispersive X-ray (EDX). The study focused on the catalytic efficiency of HyTL Al_3_Y-Rh_*x*_ in the oxidation of CO and CO-PROX. The impact of Rh enrichment on the supports of HyTL Al_3_Y samples was investigated to improve the catalyst’s performance. Thus, fundamental insights into the interaction between metals and their supports and the CO oxidation and CO-PROX processes on the HyTL Al_3_Y-Rh_*x*_ nanocatalysts have been uncovered. Yet, to the best of our knowledge, no comparative analyses have been conducted on samples leached hydrothermally regarding CO oxidation and CO-PROX catalytic activity.

## 2 Experimental Methods

### 2.1 Synthesis of Samples

Initially, intermetallic compounds’ stoichiometric amounts of relevant Al_3_Y-Rh_*x*_ (*x* = 0, 0.2, 0.5, 1.0) were weighed in a glovebox and melted in an ARC furnace under argon. Heat treatment at 900 °C for 48 h in an inert environment promotes oxidation. Continuous melting for 2 days ensured material homogeneity. Homogenization aims to soften and adapt the substance under study’s structure and features. Softer materials are far less likely to crack under stress. To minimize sample crystal dislocation, after annealing, the ingots were ground and crushed into powders under a 100-size mesh. HyTL followed the sample collection. Individually, 1 g of Al_3_Y-Rh_*x*_ (*x* = 0, 0.2, 0.5, 1.0) powder samples was dissolved in 20 wt % KOH (100 mL) solution. A homogeneous solution was achieved by stirring the mixture. After being stirred, the solution was placed in a Teflon-sealed cup and autoclaved at 130 °C for 12 h in air. After the hydrothermal reaction, the materials were filtered and washed multiple times. Additionally, centrifuging the solution with DI water adjusted the pH to 7. All subsequent analyses used well-defined materials. Scheme 1 represents the experimental setup for material synthesis and CO catalytic oxidation equipment.

### 2.2 Catalysis Measurements

In order to evaluate catalysis in a gaseous mixture of CO (0.5%) and O_2_ (0.25%)/He at 30 mL min^–1^, a standard fixed-bed flow reactor was utilized. A ball of quartz wool was used to sustain an approximately 100 mg of the sample within a straight quartz tube with an inner diameter of 6 mm. Every catalytic investigation was conducted between 298 and 772 K. Utilizing a Shimadzu GC-8A online gas chromatograph equipped with molecular sieve 5A (O_2_, CO) and Porapak Q (CO_2_) columns, the reaction products were monitored. Utilizing the percentage conversion of CO to CO_2_, the catalytic activity for CO–O_2_ oxidation was determined. The reaction attained a steady state after 30 min, at which point the peak region was determined. The conversion of CO to O_2_ was calculated utilizing eqs 1 and 2.^22^
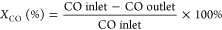
1
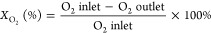
2

## 3 Results and Discussion

The spectral investigation of all of the samples provides an in-depth understanding of the nature of the materials, including their phase purity, chemical composition, and crystalline nature, both before and after the HyTL procedure occurred.^22^

### 3.1 X-ray Diffraction Analysis

**Figure 1:**
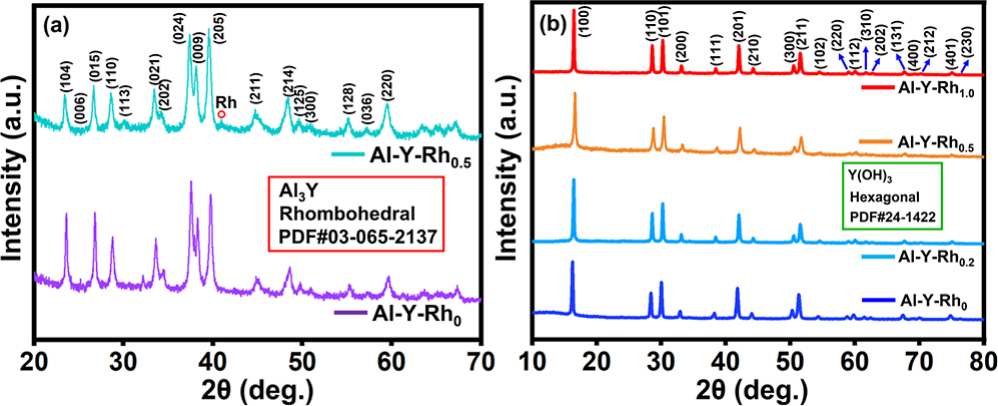
XRD patterns (a) before and (b) after HyTL of Al_3_YRh_*x*_.

The XRD analysis of Al_3_Y-Rh_*x*_ (*x* = 0, 0.2, 0.5, 1.0) samples before leaching is shown in Figure 1a. The diffractogram of both counterparts, including the rhombohedral Al_3_Y phase, is observed to co-occur. The major diffraction patterns are observed at 23.5°, 25.2°, 28.8°, 30.5°, 32.1°, 33.5°, 34.5°, 37.5°, 38.3°, 39.8°, 44.9°, 45.0°, 46.0°, 47.8°, 48.4°, 49.8°, 50.9°, 51.8°, 52.9°, 54.4°, 55.2°, 57.3°, and 59.6° values to be slightly shifted due to the lattice strains and structure relaxations. In the case of Al_3_Y-Rh_*x*_ samples, a small peak is observed at 2θ values due to the presence of a trace amount of Rh (Rh 2θ values at 41.0°; JCPDS: 01-089-7383).^28^ The XRD diffraction patterns of Al_3_Y-Rh_*x*_ (*x* = 0, 0.2, 0.5, 1.0) samples match with the Al_3_Y phase and corresponding JCPDS card no. 03-065-2137.^29^ The absence of discernible patterns in the XRD spectra proves its high purity of Al_3_Y. The XRD spectra of Al_3_Y-Rh_*x*_ (*x* = 0, 0.2, 0.5, 1.0) samples after HyTL are shown in Figure 1b. It exhibits the exact diffractogram patterns at 16.2°, 28.4°, 30.0°, 32.8°, 38.2°, 41.7°, 44.0°, 50.3°, 51.2°, 54.4°, 58.7°, 59.8°, 61.5°, 62.4°, 67.4°, 69.9°, and 74.8° of the Y(OH)_3_ phase. The XRD diffractograms of the HyTL samples are in agreement with the hexagonal structure of JCPDS card no. 01-083-2042.^29^ The crystallographic values of Y(OH_3_) obtained from TOPAS software and the Scherrer equation are shown in Table 1.

**Table 1:** Calculated Values and Other Crystallographic Values of Prepared Samples

samples	space group	crystal system	a (Å)	b (Å)	c (Å)	*D*_p_ (nm)
*x* = 0	*P*63/*m*	hexagonal	6.16	6.16	3.52	57.08
*x* = 0.2			6.22	6.22	3.53	48.55
*x* = 0.5			6.27	6.27	3.55	39.70
*x* = 1.0			6.26	6.26	3.54	43.67

### 3.2 Morphological and Elemental Analysis

**Figure 2:**
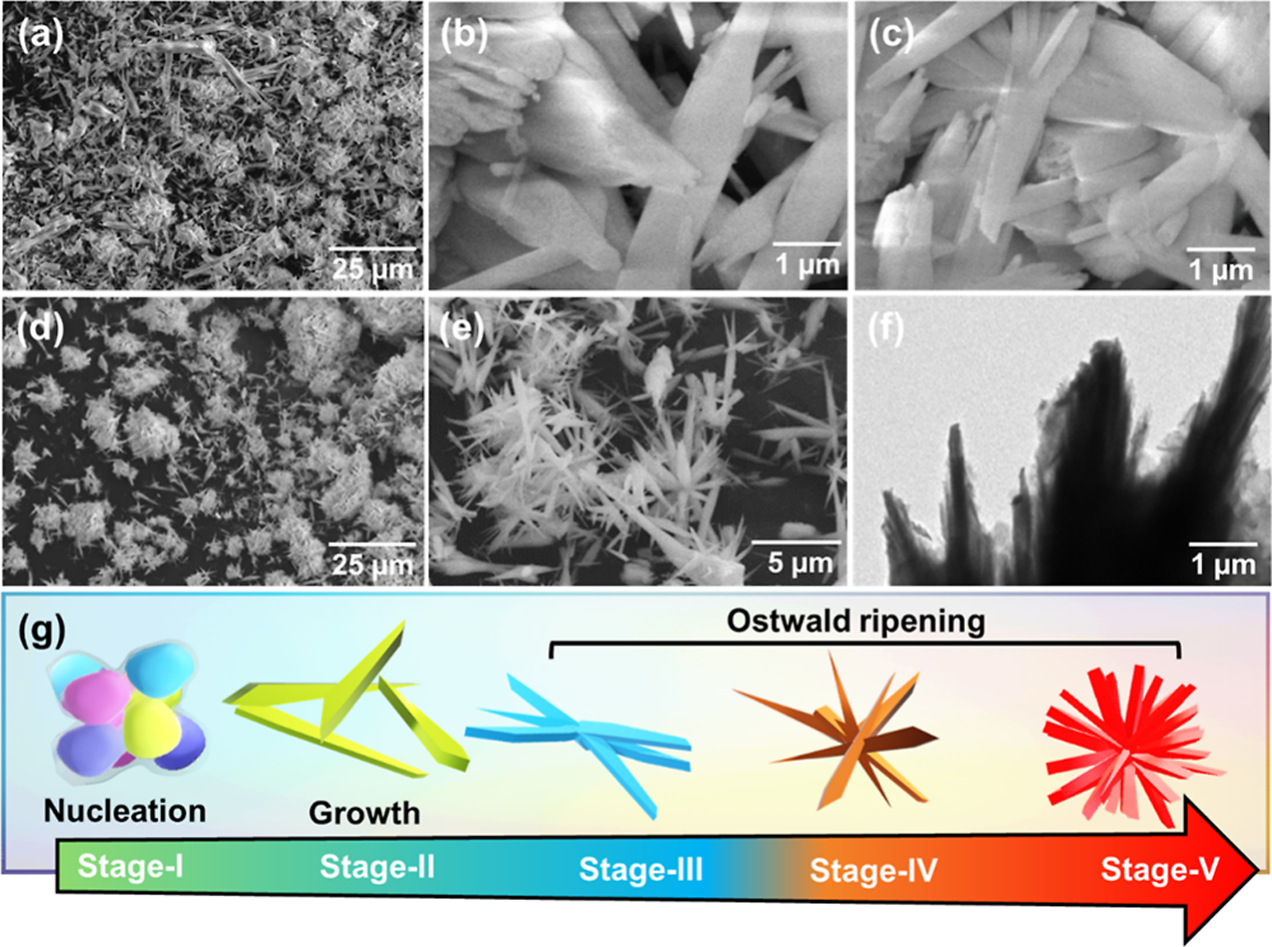
FE-SEM images of (a–c) HyTL Al_3_Y and (d,e) HyTL Al_3_Y-Rh_*x*_. (f) TEM image of HyTL Al_3_Y-Rh_*x*_. (g) The possible graphic illustration of the urchin-like crystal growth mechanism of Al_3_Y-Rh_*x*_ is step-by-step.

The SEM analysis technique was utilized to gain knowledge about the prepared samples’ topological and 3D morphological characteristics. The SEM images of HyTL Al_3_Y in Figure 2a–c revealed random needlelike irregular rod-shaped particles. Similarly, the SEM and TEM images of HyTL Al_3_Y-Rh_*x*_ as shown in Figure 2d–f expose similar needle morphology simulating urchin-like structures, which could account for the addition of Rh in the sample. Thus, these planes further support the XRD results. Further, to affirm the presence of the elements in leached Al_3_Y and Al_3_Y-Rh_*x*_ samples, EDX analysis was carried out on the prepared materials. With Ostwald’s ripening of the Al_3_Y-Rh_*x*_ urchin-like structure demonstrated step-by-step, the feasible graphic illustration of the urchin-like crystal formation mechanism is presented in Figure 2g. As depicted in Figure S1a,b, the elemental analysis recorded in six different places at the Al_3_Y surface revealed uniformly distributed atomic % of elements Y (24.24%) and O (75.55%), which contains trace amount of Al (0.21%). Further, the elemental analysis in six different places at the Al_3_Y-Rh_*x*_ surface revealed uniformly distributed Y (21.70%), O (77.68%), and Rh (0.35%)^30^ with the trace amount of Al (0.25%) as exhibited in Figure S1c,d.

### 3.3 Catalytic Activity Assessment on CO Oxidation and CO-PROX

**Figure 3:**
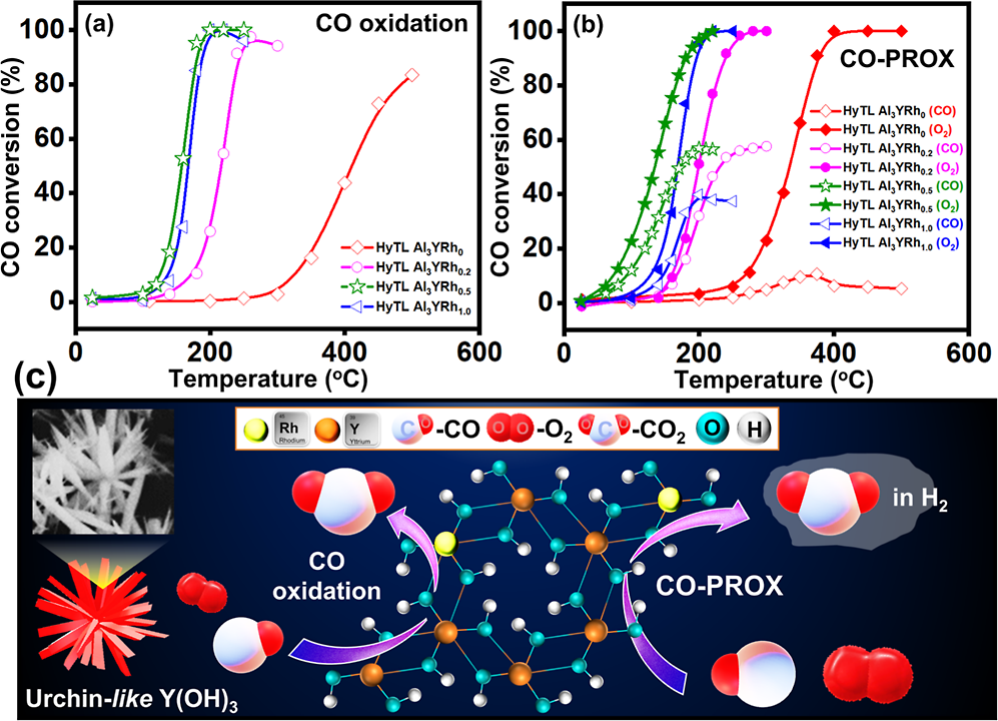
Catalytic activity of CO oxidation at (a) HyTL Al_3_Y-Rh_*x*_ (*x* = 0, 0.2, 0.5, 1.0) and CO-PROX at (b) HyTL Al_3_YRh_*x*_ (*x* = 0, 0.2, 0.5, 1.0). (c) Schematic illustration of the Mars–van Krevelen mechanism of oxidation of CO.

In the processes of CO oxidation (Figure 3a) and CO-PROX (Figure 3b), the catalytic activity of HyTL Al_3_Y-Rh_*x*_ (*x* = 0, 0.2, 0.5, 1.0) was observed. Each of the samples that were used was obtained using HyTL. In Figure 3a,b, it is observed that the HyTL treatment and the presence of Rh extensively improved the catalytic performance of the leached set of Al_3_Y-Rh_*x*_ (*x* = 0.2, 0.5, 1.0) samples in comparison to the leached Al_3_YRh_0_ sample with respect to the CO oxidation and CO-PROX reactions. On the other hand, one of the most notable aspects of this study in CO-PROX is that the amount of CO that is the conversion by HyTL Al_3_Y-Rh_*x*_ catalysts does not decrease. Despite a significant quantity of H_2_, this suggests that oxygen selectively interacts with CO. It possesses many desirable characteristics. Rh particles on the surface of Y(OH)_3_ are primarily responsible for the enhanced catalytic performance of HyTL Al_3_Y-Rh_0.5_. Compared with the other investigated samples, HyTL Al_3_Y-Rh_0.5_ demonstrates enhanced CO catalytic activity. A range of reasons are responsible for this increased rate of CO oxidation and CO-PROX, including the following: The observation that Al_3_Y-Rh_0.5_ has a smaller average crystallite (grain) size than other samples, Y(OH)_3_ redox activity is probably connected with this characteristic. In order to catalyze oxidation reactions that involve CO oxidation and CO-PROX, it is necessary to have redox reactions that involve changes in the oxidation states of yttrium and oxygen. It is simple for reactant molecules to be adsorbed and activated when there is a higher concentration of oxygen vacancies. Rh and Y(OH)_3_ interact with one another. These characteristics are highly advantageous for the oxidation of the CO and CO-PROX reactions. It also helps activate oxygen on the surface of Y(OH)_3_, providing a typical synergy platform for the oxidation of CO and CO-PROX. In addition to situating the undiffused atom, the HyTL impact is responsible for stimulating the synergy between Rh and Y(OH)_3_. The Rh particles mentioned above supply HyTL Al_3_Y-Rh_0.5_ with many active sites. The stated HyTL-Al_3_Y-Rh_0.5_ catalyst presents notable benefits in CO oxidation owing to its distinctive synthesis and material characteristics. The HyTL procedure enables the efficient distribution of Rh throughout the Y(OH)_3_ matrix, leading to robust metal-support interactions. These interactions stabilize the Rh active sites and augment catalytic efficiency. Furthermore, the interaction between Rh and the leached Al_3_Y intermetallics enhances the occurrence of oxygen vacancies, which are essential for augmenting oxygen mobility and expediting the oxidation process, particularly at reduced temperatures. The catalyst has strong efficacy in CO oxidation and CO-PROX processes, facilitated by an improved aggregation-activation mechanism. This ensures enhanced catalytic activity and selectivity, positioning the material as a viable option for low-temperature CO oxidation and CO-PROX reactions and sustainable environmental applications.

### 3.4 Mars–van Krevelen Mechanism

The Mars–van Krevelen mechanism leads to CO catalytic oxidation over the HyTL Al_3_Y-Rh_0.5_ catalyst (Figure 3c). The mechanism involves the generation of CO_2_ by interacting with the tangled surface lattice atom of the O_2_ atom from the Rh–Y(OH)_3_ platform. CO desorbs oxygen from the catalyst, resulting in the formation of an oxygen vacancy. Consequently, the subsequent step necessitates an additional reaction with molecular oxygen to occupy the oxygen vacancy. After restoring the oxygen vacancy, the following CO molecule undergoes oxidation until the Rh–Y(OH)_3_ interface is established, facilitating the fast oxidation of the subsequent CO molecule.^31^ Thus, Rh and Y(OH)_3_ are crucial for the catalyst interface formation. HyTL of the catalyst enhances the quantity of Y(OH)_3_ supports while the deposited Rh occupies minor voids or voids produced by the supports. Consequently, the HyTL Al_3_Y-Rh_0.5_ catalyst exhibits efficacy in the CO oxidation and CO-PROX reactions.

### 3.5 X-ray Diffraction Analysis after CO Oxidation

**Figure 4:**
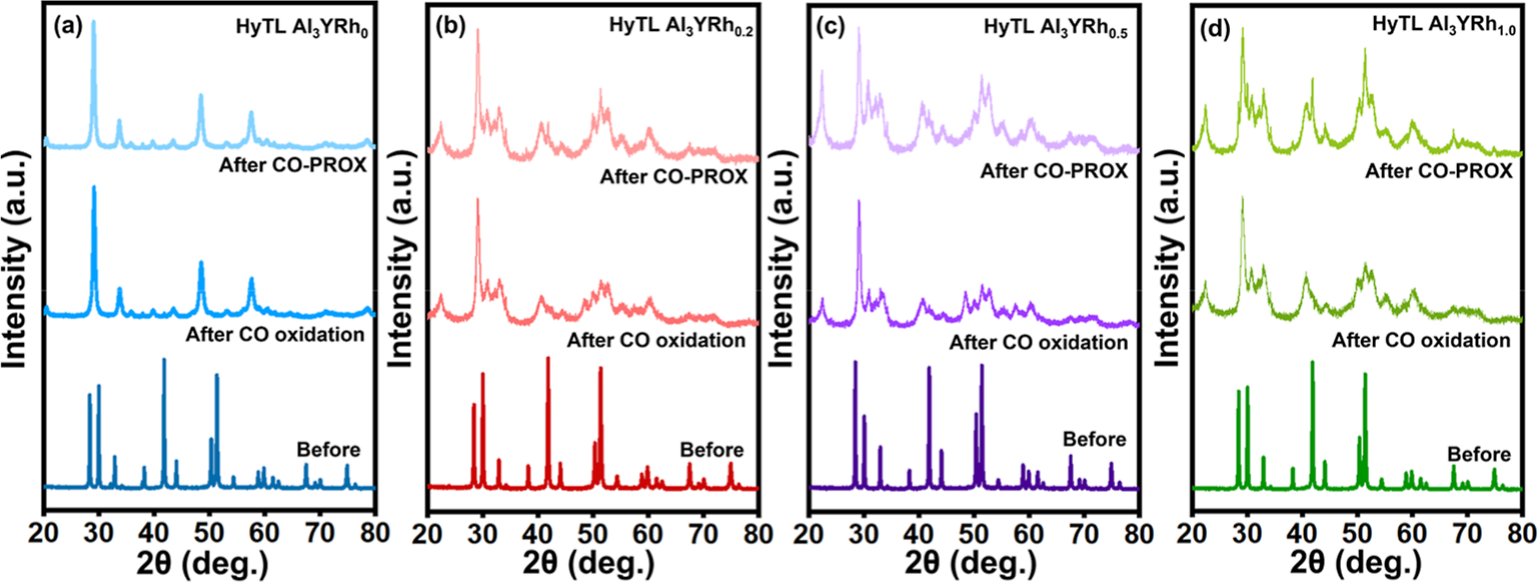
XRD patterns of before and after CO oxidation and CO-PROX reactions of (a) HyTL Al_3_YRh_0_, (b) HyTL Al_3_YRh_0.2_, (c) HyTL Al_3_YRh_0.5_, and (d) HyTL Al_3_YRh_1.0_.

XRD analysis was used to examine the crystalline characteristics of the materials in their prepared states for both compositions after the catalytic process was completed (Figure 4a–d). After that, the diffraction patterns of the samples of (b) HyTL Al_3_YRh_0.2_, (c) HyTL Al_3_YRh_0.5_, and (d) HyTL Al_3_YRh_1.0_ were observed to exhibit the patterns of yttrium oxide hydroxide (YOOH) (JCPDS no.: 00-020-1413)^32^ and rhodium oxide (Rh_2_O_3_) (JCPDs no.: 00-043-0009)^33,34^ phases. This was observed following catalytic CO oxidation and CO-PROX reactions. On the other hand, the HyTL Al_3_YRh_0_ catalyst revealed a phase of yttrium oxide (Y_2_O_3_) (JCPDs no.: 00-020-1412).^32,35^ The results obtained correspond to the JCPDS card numbers associated with each individual. All of the results of the XRD study are presented clearly and concisely in Figure 4a–d.

## 4 Conclusion

In conclusion, we rationally developed highly potential Rh–Y(OH)_3_ nanocatalysts via HyTL of intermetallic compounds with the composition Al_3_YRh_*x*_ (*x* = 0, 0.2, 0.5, 1.0) at 130 °C for 12 h in 20 wt % KOH. The structural characterization investigation included all synthesized catalysts. The results showed good structural purity, low-temperature reduction, excess positively charged Rh species, reduced crystallite sites, chemical valence state, and lower crystallite (grain) size. After HyTL, the synergic interaction between the Rh–Y(OH)_3_ linkage and active sites reduced CO oxidation and CO-PROX reaction barriers. It improved HyTL Al_3_Y-Rh_0.5_ catalytic behavior compared with other samples. The HyTL Al_3_Y-Rh_0.5_ catalyst exhibits good catalytic activity due to its architecture, including porosity, particle size dispersion, and thermal stability. This study shows the importance of structure–activity connections in creating thermally stable and highly active catalysts for CO oxidation and CO-PROX. This motivates and facilitates the development of next-generation durable catalysts to clean hazardous CO gas emissions. Catalytic CO conversion produces CO_2_ gas for fuel production and automobile pollution regulation.

## Supporting Information Available

Details of instrumentation and methods and EDX atomic percentage of after HyTL Al_3_Y and Al_3_YRh_*x*_ catalysts (PDF)
