# Supplementary material for: Selective Hydrothermal Leaching of Aluminum from Al3YRhx (x = 0, 0.2, 0.5, 1.0) Intermetallic Compounds: The Effect of Rh Variants in Comparing the Catalytic CO Oxidation and CO-PROX Reactions
Source: ACS Mater Au. 2024 Nov 29;5(3):502–7. doi: 10.1021/acsmaterialsau.4c00140 (PMC12082349; doi:10.1021/acsmaterialsau.4c00140)
Supplement: Supplementary file 1 — mg4c00140_si_001.pdf [file mg4c00140_si_001.pdf]

Supporting information

Selective Hydrothermal Leaching of Aluminum from Al_3YRh_x (x=0,0.2,0.5,1.0) Intermetallic Compounds: The Effect of Rh Variants in Comparing the Catalytic CO Oxidation and CO-PROX Reactions

Balasubramanian Sriram[a], Sea-Fue Wang[a], Satoshi Kameoka[b]**

[a]*Department of Materials and Mineral Resources Engineering, National Taipei University of Technology, No. 1, Section 3, Chung-Hsiao East Road, Taipei 106, Taiwan, ROC.*

[b]*Institute of Multidisciplinary Research for Advanced Materials, Tohoku University, 2-1-1 Katahira, Aoba-ku, Sendai, 980-8577, Japan.*

Corresponding authors

S.F. Wang: sfwang@ntut.edu.tw

S. Kameoka: satoshi.kameoka.b4@tohoku.a.jp

No. of pages: 3

No. of figures: 1

Table of Content Entry	Page No.
Instrumentation and methods	S2
**Figure S1. (a-d)** EDX atomic percentage of after HyTL Al_3Y and Al_3YRh_x catalyst	S3

Instrumentation and methods

The microstructure and the elemental composition of the as-prepared materials were studied employing a high resolution (HR) transmission electron microscope (TEM) (JEOL JEM-2100F (HR)) operating at 200 kV and by energy-dispersive X-ray spectroscopy using EDAX AMETEK Inc., DigitalMicrograph® software and scanning electron microscopy (SEM, HITACHI, Regulus 8100). Phase configuration is identified using Bruker (XRD, 2D phaser). By utilizing these characterization methods, thephysical properties of the prepared materials are investigated. The reaction products were monitored by an on-line gas chromatograph (Shimadzu GC-8A) equipped with molecular sieve 5A (O_2, CO) and Porapak Q (CO_2) columns.

(a)

Area	Y	O	Al
1	21.87	77.91	0.23
2	26.93	72.8	0.26
3	25.54	74.21	0.26
4	23.35	76.46	0.18
5	22.12	77.73	0.15
6	26.12	73.66	0.22
Avrg.	24.24	75.55	0.21

(b)

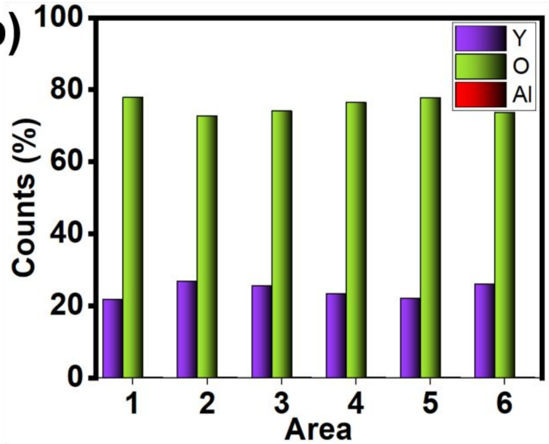

(c)

Area	Y	O	Rh	Al
1	21.87	77.91	2.97	0.23
2	26.93	72.8	0.22	0.26
3	25.54	74.21	0.08	0.26
4	23.35	76.46	0.09	0.18
5	22.12	77.73	0.08	0.15
6	26.12	73.66	0.00	0.22
Avrg.	21.70	77.68	0.35	0.25

(d)

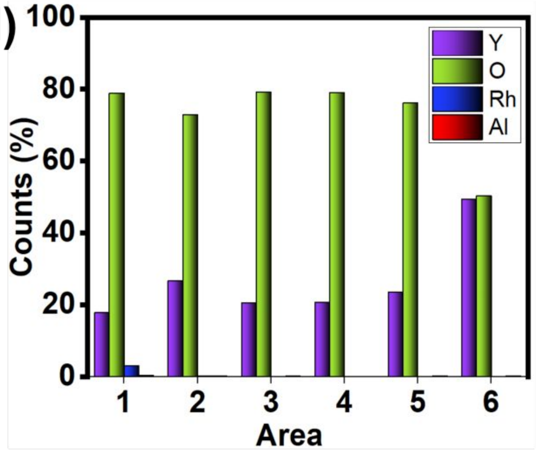

Figure S1. (a-d) EDX atomic percentage of after HyTL Al_3Y and Al_3YRh_x catalyst.
